# The Use of a Synthetic Hybrid-Scale Fiber Matrix to Treat Difficult-to-Heal Wounds

**DOI:** 10.7759/cureus.50405

**Published:** 2023-12-12

**Authors:** Melanie Wilson

**Affiliations:** 1 Operations, Chronic Wound Solutions of Texas, San Antonio, USA

**Keywords:** wound healing, hybrid-scale fiber matrix, synthetic, lower extremity wounds, post mohs wound, difficult-to-treat wounds

## Abstract

Introduction: Complex and chronic wounds are often difficult to treat, and current advanced therapies have their limitations. A synthetic hybrid-scale fiber matrix could be a viable option in treating these wounds, as previous clinical studies utilizing the matrix have shown positive results in treating chronic ulcers and surgical wounds.

Methods: Patients with difficult-to-treat wounds of varying etiologies were treated with a synthetic hybrid-scale fiber matrix (Restrata®, Acera Surgical, Inc., St. Louis, Missouri). The wound bed was debrided and prepared, and the synthetic matrix was prepared and applied to the wound. Wounds were monitored for healing progress. Additional applications of the synthetic matrix were used based on clinician discretion.

Results: Six patients with wounds of varying etiologies were assessed, including a 30-year recalcitrant wound. All wounds achieved significant healing, with four of the six wounds (67%) achieving complete closure in an average of 57.8 ± 27.0 days (8.3 ± 3.9 weeks).

Conclusions: The study found that the synthetic matrix was effective, resulting in improved healing across various etiologies, including cancer resection and amputation. The clinical results presented here suggest that the synthetic hybrid-scale fiber matrix may be an optimal alternative in treating difficult-to-heal wounds.

## Introduction

Wounds of varying etiologies, including chronic diabetic foot ulcers, venous leg ulcers, post-Mohs wounds, and surgical wounds, can be difficult to heal [1]. Various solutions have been utilized to treat wounds of these etiologies, including autografts, allografts, and xenografts [2]. However, these modalities have multiple limitations. Complications associated with biologic matrices include limited availability, risk of inflammatory response, and disease transmission, while autografting introduces the risk of donor site morbidity [2]. Additionally, biologic wound matrices present challenges to hospital systems by requiring tissue tracking and, in some cases, special storage [2].

A synthetic hybrid-scale fiber matrix (Restrata®, Acera Surgical, Inc., St. Louis, Missouri) offers a new solution for difficult-to-treat wounds [3]. This matrix is composed of two biocompatible polymers, polyglactin 910 and polydioxanone, electrospun into a porous structure with hybrid-scale fiber diameters [3]. The hybrid-scale fiber matrix provides a scaffold with an architecture similar to the native human extracellular matrix, without the risks of disease transmission or inflammation associated with biological materials [3,4]. The size and structure of the synthetic hybrid-scale fiber matrix encourage cellular ingrowth and neovascularization [3]. Engineered to resorb via hydrolysis over a period of approximately one to four weeks [4-6], it matches the tissue ingrowth and provides mechanical offloading from the matrix to the newly formed tissue [3]. The matrix's pore size ranges from 1 to 500 μm, and as it resorbs, its porosity increases, allowing for cellular differentiation within the matrix and further neovascularization [3]. Published clinical outcomes have demonstrated that treatment with this synthetic matrix resulted in the successful healing of various wounds, including venous ulcers, pressure ulcers, neuropathic foot ulcers, and complex traumatic and surgical wounds [5-9].

Given the encouraging clinical results published to date, the objective of the present retrospective case series was to evaluate the clinical efficacy of the hybrid-scale fiber matrix for difficult-to-treat wounds. Select cases were presented at the Symposium on Advanced Wound Care Fall virtual conference from November 4-6, 2020, as a poster presentation.

## Materials and methods

In this retrospective case series, patients with difficult-to-treat wounds of varying etiologies were treated with a synthetic hybrid-scale fiber matrix (Restrata®, Acera Surgical, St. Louis, Missouri). Data were collected through a retrospective review of medical records from 2019 to 2020. The study was exempt from institutional review board approval due to the absence of patient-identifying information. All subjects provided written informed consent. To be included in this series, patients must have received at least one application of the synthetic matrix. A total of six patients were identified for inclusion. These individuals opted for the synthetic fiber matrix over split-thickness skin grafting. A majority presented with long-standing wounds, wounds that had failed or stalled with prior advanced treatments, or a history of wound healing complications. Data on age, sex, prior comorbidities, and previous wound treatments were collected for each patient, along with wound-specific details such as etiology, location, and age.

For all cases, the wound bed was debrided with a curette to viable tissue to ensure full contact with the synthetic matrix. The matrix was cut to wound size, fenestrated with a scalpel, and secured to the wound bed with adhesive strips. An absorbent, non-adherent primary dressing was placed over the matrix. Hydrogel was applied to dry wounds as needed, covered with a dry dressing. Wounds were monitored for healing progress as clinically indicated. At each visit, the wound was debrided as necessary, and its size measured. Additional applications of the synthetic matrix were used based on clinician discretion. Wounds were considered fully healed once they were completely re-epithelialized and the wound site was closed.

## Results

Six patients with wounds of varying etiologies were included in the present case series (Table 1). All wounds achieved significant healing. Overall, 67% (four of six) of the wounds achieved complete closure in an average of 57.8 ± 27.0 days (8.3 ± 3.9 weeks).

**Table 1:** Summary of cases TMA = transmetatarsal amputation, VLU = venous leg ulcer, ​​​​​​​DFU = diabetic foot ulcer, ​​​​​​​NPWT = negative pressure wound therapy, ​​​​​​​STSG = split-thickness skin graft, ​​​​​​​HBOT = hyperbaric oxygen therapy

Demographics and Wound Characteristics	Patient 1	Patient 2	Patient 3	Patient 4	Patient 5	Patient 6
Initial wound size (cm)	9.5 × 12.5 × 1.2	7.2 × 3.6 × 0.3	3 × 4 × 0.3	2.9 × 2 × 0.3	5.1 × 5.2 × 0.3	2 × 3 × 0.2
Wound etiology	TMA	Toe and metatarsal head amputation	Scleroderma/squamous cell carcinoma resection	Post-Mohs	VLU	DFU
Patient age (years)	55	44	70	35	74	69
Age of wound (weeks)	10	4	1,560	4	8	4
Prior failed wound therapies	NPWT,collagenase ointment	NPWT	STSG,HBOT	none	Collagen, NPWT	Calcium alginate
Wound closure achieved	No	Yes	Yes	No	Yes	Yes
Time to heal (weeks)	NA	11	6	NA	12	4
Number of synthetic hybrid-scale fiber matrix applications	7	5	5	9	4	4

Case 1: transmetatarsal amputation wound

**Figure 1:**
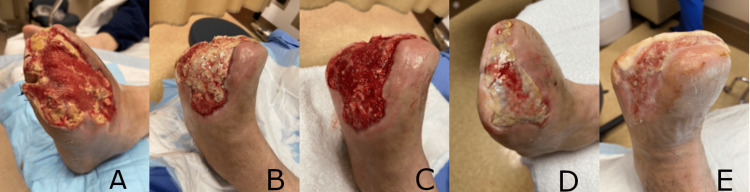
Progressive and significant healing of a transmetatarsal amputation wound following seven weekly applications of the synthetic matrix over 10 weeks. (A) Transmetatarsal amputation wound prior to initial application of the synthetic hybrid-scale fiber matrix, measuring 9.5 cm long x 12.5 cm wide x 1.2 cm deep. (B) Transmetatarsal amputation wound at week 1. The synthetic hybrid-scale fiber matrix can be seen resorbing into the wound bed. (C) Transmetatarsal amputation wound at week 2, with the wound bed demonstrating granulation tissue formation. (D) Continued healing of the transmetatarsal amputation wound at week 6.  (E) Transmetatrsal amputation wound at week 10, demonstrating re-epithelializiation and significant decrease in overall wound size.

A 55-year-old male with type II diabetes mellitus presented with gas gangrene in the left fourth toe. The patient's computed tomography scan revealed subcutaneous gas and soft tissue swelling in the left forefoot, with erosive changes in the distal phalanx of the fourth toe suggestive of osteomyelitis. He was started on antibiotics and underwent a transmetatarsal amputation (TMA). The TMA wound initially measured 20 cm long x 10 cm wide x 0.6 cm deep, with exposed tendon and necrotic adipose tissue. The wound was initially treated with negative pressure wound therapy (NPWT) and collagenase ointment, resulting in a reduction of the wound size to 9.5 cm x 12.5 cm after 10 weeks of treatment (Figure 1A). However, the wound healing then stalled, necessitating the application of an alternative advanced modality. The wound underwent serial debridements, hyperbaric oxygen therapy (HBOT), and multiple applications of a synthetic hybrid-scale fiber matrix as needed. Over 10 weeks, with seven weekly applications of the material, the wound demonstrated significant healing. This healing was evidenced by a decrease in wound size and depth, formation of granulation tissue, and re-epithelialization (Figures 1B-E). The patient was lost to follow-up before complete healing was achieved.

Case 2: toe and metatarsal head amputation wound

**Figure 2:**
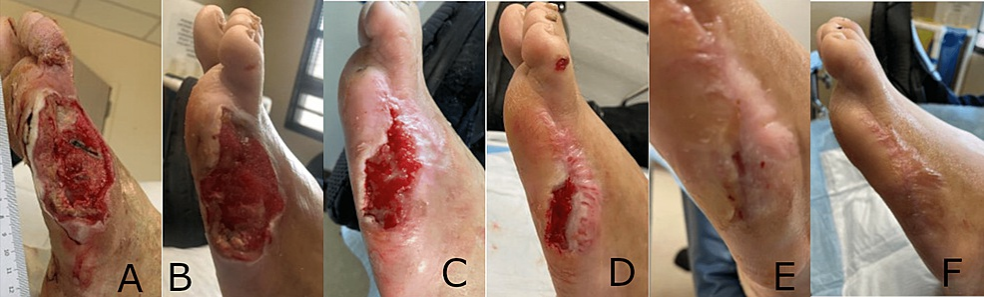
Progressive healing of a toe and metatarsal head amputation wound following five applications of the synthetic hybrid-scale fiber matrix, resulting in complete closure and healing of the wound after 11 weeks. (A) Amputation wound prior to the initial application of the synthetic hybrid-scale fiber matrix. (B) Regranulation of the wound bed observed one week after the initial application of the synthetic hybrid-scale fiber matrix, measuring 6.8 cm x 3.5 cm x 0.2 cm. (C) Continued healing of the toe and metatarsal head amputation wound observed at week 2 following two applications, measuring 6.3 cm x 2.9 cm x 0.2 cm. (D) Notable regranulation and re-epithelialization wound at week 3 following three applications. (E) Further re-epithelialization of the amputation wound observed at week 7 following five applications. (F) Complete closure observed at 11 weeks following five applications of the synthetic hybrid-scale fiber matrix.

A 44-year-old male with type II diabetes mellitus and neuropathy presented with a Wagner Grade 3 diabetic foot ulcer (DFU) to the left lateral foot and fifth metatarsal head. The wound failed to heal following serial debridements and progressed to a Wagner Grade 4 DFU with osteomyelitis. The patient underwent a left fifth toe and metatarsal head amputation. The surgical wound, which had exposed bone, was originally treated with NPWT. NPWT was later discontinued, the wound was debrided to 7.2 cm long x 3.6 cm wide x 0.3 cm deep one month after the amputation (Figure 2A), and the synthetic hybrid-scale fiber matrix was applied. Serial debridements and multiple applications of the synthetic hybrid-scale fiber matrix were utilized as appropriate to stimulate healing. Progressive wound healing and formation of granulation tissue were observed during treatment with the synthetic matrix with no signs of infection. After five applications over 11 weeks, the wound achieved 100% epithelialization and was completely healed (Figure 2B-F).

Case 3: scleroderma and squamous cell carcinoma wound

**Figure 3:**
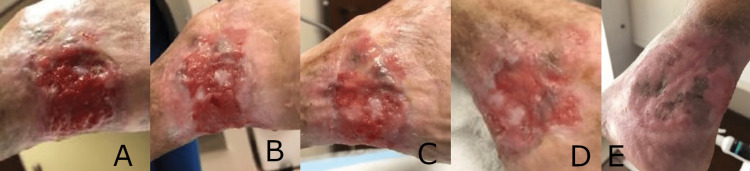
Healing progress of a 30-year recalcitrant ankle wound following five applications with the hybrid-scale fiber matrix, resulting in complete healing after six weeks. (A) Recalcitrant ankle wound with a history of poor healing following treatment with hyperbaric oxygen therapy (HBOT) and prior to initiation of the synthetic hybrid-scale fiber matrix. (B) Re-granulation observed in the wound bed one week after application of the synthetic hybrid-scale fiber matrix. (C) Continued healing of the ankle wound observed at week 3 following two applications. (D) A significant decrease in wound size and re-epithelialization of the wound observed at week 4. (E) Complete closure of the 30-year recalcitrant ankle wound observed at week 6 after five applications of the synthetic hybrid-scale fiber matrix.

A 70-year-old female with scleroderma and a recurrent squamous cell carcinoma presented with a wound on the left posterior ankle that was over 30 years old. The squamous cell carcinoma was excised, and wound margins were confirmed negative for tumor involvement by the dermatologist prior to referral for wound management. The patient had failed previous treatments, including skin grafting. Due to the patient’s poor wound healing progression over the past 30 years, as well as complicating factors such as impaired mobility and local infection, staging of care was implemented. The wound was initially treated with HBOT. After 24 HBOT treatments, the synthetic hybrid-scale fiber matrix was initiated. After discontinuation of HBOT and prior to initial application of the synthetic hybrid-scale fiber matrix, the wound measured 3.5 cm long x 4 cm wide x 0.3 cm deep (Figure 3A). After five matrix applications over six weeks, the 30-year recalcitrant wound demonstrated re-epithelialization and complete healing (Figure 3B-E).

Case 4: post-Mohs wound

**Figure 4:**
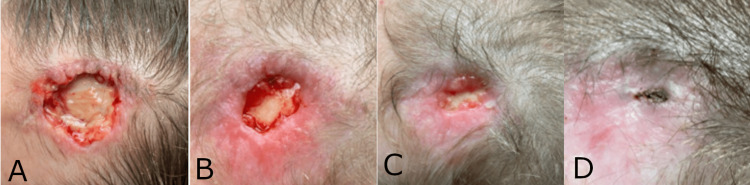
Progressive healing of a post-Mohs wound following nine applications using the synthetic hybrid-scale fiber matrix over the course of 10 weeks. (A) Initial presentation of the post-Mohs wound prior to the application of the synthetic hybrid-scale fiber matrix. (B) Granulation tissue formation and a decrease in wound size observed at week 6 following five applications. (C) A wound demonstrating re-epithelialization and a significant decrease in size at week 8 after seven applications. (D) Significant reduction in overall wound size 10 weeks after initial treatment with the synthetic hybrid-scale fiber matrix, with a total of nine applications.

A 35-year-old female with a history of tobacco use presented with a full-thickness tissue defect and exposed calvarium covered with yellow biofilm. The patient had previously undergone a Mohs procedure for basal cell carcinoma over her left forehead and acquired a Staphylococcus infection in the post-surgical period, leading to abscess formation and wound dehiscence. The Staphylococcus infection was treated with systemic antibiotics. Four weeks after the Mohs procedure, the wound was debrided to 2.9 cm long x 2 cm wide x 0.3 cm deep (Figure 4A), and the synthetic hybrid-scale fiber matrix was applied and secured using Steri-Strips. After nine applications over 10 weeks, notable healing was observed, including the formation of new granulation tissue and a significant reduction in wound size (Figure 4B-D). The patient was lost to follow-up prior to complete healing.

Case 5: venous leg ulcer

**Figure 5:**
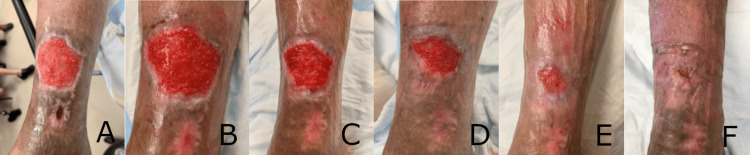
Healing progress of a venous leg ulcer following four applications with the hybrid-scale fiber matrix, resulting in full wound closure after 12 weeks. (A) Venous leg ulcer prior to application of the synthetic hybrid-scale fiber matrix. (B) Venous leg ulcer one week after initial application of the synthetic hybrid scale fiber matrix. (C) Venous leg ulcer two weeks after initial application of the synthetic hybrid scale fiber matrix, with a 33% decrease in wound size. (D) A continued decrease in overall wound size of the venous leg ulcer. (E) Continued re-epithelialization and a wound area decrease of the venous leg ulcer. (F) Complete wound closure 12 weeks after initial application of the synthetic hybrid-scale fiber matrix.

A 74-year-old male with a history of type II diabetes mellitus with neuropathy, peripheral vascular disease, and coronary artery disease presented with a venous leg ulcer refractory to other treatment, including a collagen material and NPWT. The patient had previously undergone a left popliteal balloon angioplasty that led to increased pain and ischemia with gangrene and eschar to the tibial area. The patient then underwent a left femoral thrombectomy and endarterectomy, profundaplasty, and left distal superficial femoral artery to tibioperoneal trunk bypass with reverse great saphenous vein. The ulcer was debrided of eschar and necrotic tissue and treated with collagen particles and NPWT. However, the wound failed to heal after eight weeks, so it was then debrided to an initial size of 5.1 cm long x 5.2 cm wide x 0.3 cm deep and treated with the synthetic hybrid-scale fiber matrix (Figure 5A). After four applications over 12 weeks, the wound achieved full closure (Figures 5B-F).

Case 6: diabetic foot ulcer

**Figure 6:**
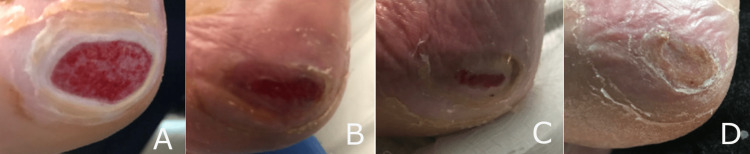
Progressive healing of a diabetic heel wound following four applications with the hybrid-scale fiber matrix, resulting in complete wound healing after four weeks. (A) Diabetic heel wound prior to the first application of the synthetic hybrid-scale fiber matrix. (B) Diabetic heel wound after two applications of the synthetic hybrid-scale fiber matrix, demonstrating a notable decrease in wound area. (C) Diabetic heel wound at week 3 showing continued re-epithelialization. (D) Complete wound closure four weeks after the initial application of the synthetic hybrid-scale fiber matrix.

A 69-year-old male with type II diabetes mellitus was referred for evaluation of a left lateral heel DFU Wagner Grade 1, which had been present for more than four weeks and had failed standard of care and calcium alginate treatment. The synthetic hybrid-scale fiber matrix was fenestrated and applied to the wound bed, which measured 2.0 cm long x 3.0 cm wide x 0.2 cm deep (Figure 6A), using Steri-Strips after wound debridement and site preparation. After four applications over four weeks, the DFU achieved complete re-epithelialization and full wound closure (Figure 6B-D). The patient also reported a reduction in pain in the wound and foot over the course of treatment.

## Discussion

The present retrospective case series was conducted to evaluate the clinical outcomes of difficult-to-heal wounds treated with a synthetic hybrid-scale fiber matrix. The study found that the synthetic matrix was effective for a variety of etiologies, including a recalcitrant cancer resection wound and amputations. All wounds achieved significant healing, with 67% achieving complete closure in an average of 8.3 weeks.

Treatment following the use of the hybrid-scale fiber matrix resulted in significant healing of difficult-to-heal wounds. Such wounds can result from disease, trauma, or surgery and commonly feature prolonged healing times, inflammation, recurrent infections, and a lack of significant response to conventional treatments [1,10]. In one case, a recalcitrant wound, present for 30 years and resistant to prior therapies including skin grafting, demonstrated rapid and complete healing after six weeks of treatment with the synthetic matrix. The effectiveness of the synthetic hybrid-scale fiber matrix with long-standing wounds has been demonstrated in previous studies. For instance, a retrospective study of 20 patients with diabetic foot ulcers and venous leg ulcers reported that 78% of wounds achieved complete closure [11]. The average wound age in this study was 16 months, with many patients having failed multiple advanced therapies [11].

In another case involving a wound with exposed bone, wounds presenting exposed underlying structures, such as tendons, cartilage, and bone, pose a particular challenge due to the devascular nature of these structures, compounded by potential complications like deep infections, excessive exudate, and tissue maceration [12,13]. Achieving rapid granulation over these exposed structures is critical due to the morbidity associated with such wounds. In this study, the wound with exposed bone, when treated using the synthetic matrix, resulted in rapid and successful formation of new granulation tissue over the exposed structure within 10 weeks. This outcome echoes previous cases where the synthetic hybrid-scale fiber matrix facilitated granulation tissue formation over devascular structures. For example, in the case of a 16-year-old pressure ulcer, granulation tissue formed over exposed L4 and L5 vertebral segments after 11 weeks of treatment with the matrix [5]. These healing rates are comparable to other advanced wound therapies. In a large cohort study, wounds of varying etiologies with exposed structures were treated with a bioactive human skin allograft [12]. In this study, wounds achieved full closure in an average of 15.38 weeks, starting from an average wound size of 51.37 cm^2^ [12].

As for the management of the recalcitrant cancer resection wound, the synthetic hybrid-scale fiber matrix presents a unique wound healing alternative in the post-oncologic resection setting. The synthetic hybrid-scale fiber matrix does not contain growth factors or biological agents as found in amniotic products [3]. Growth factors have an influence on how cells differentiate and may induce the activation of growth-promoting pathways in cancer cells [14]; therefore, the selection of appropriate wound modalities in this setting is critical.

Positive results similar to the ones observed in the present retrospective case series have been observed in prior case series’ utilizing the synthetic hybrid-scale fiber matrix for chronic wounds. [5,15-16]. Authors utilizing the synthetic hybrid-scale fiber matrix in lower extremity wounds have observed healing rates ranging from 75%-96% at around 12 weeks of treatment, and 100% healing in 7.9 weeks in a study of four post-Mohs surgical wounds [6]. The consistent results seen when utilizing the synthetic hybrid-scale fiber matrix across a variety of wound etiologies are likely due in part to the design of the matrix [3]. The synthetic matrix is designed to mimic native human extracellular matrix in both size and structure, and as such allows for cellular ingrowth and proliferation while resorbing at a rate tailored to match that of new tissue formation [3]. The synthetic hybrid-scale fiber matrix presented here is the first of its kind available for human clinical use [17].

The clinical results presented here suggest that the synthetic hybrid-scale fiber matrix may be an optimal alternative in the treatment of difficult-to-heal wounds. However, there are limitations to the study, including the small sample size and varying wound etiologies. The retrospective design of the study has its limitations as well, such as recall and selection bias [18]. To further confirm the results observed here, additional research should be conducted.

## Conclusions

Long-standing and complex wounds can be difficult to treat, and existing treatment modalities have their limitations. These wounds can become especially difficult to heal in the presence of multiple co-morbidities. A fully synthetic matrix may provide an alternative option capable of improving treatment and healing outcomes for these wounds.

The present case series demonstrates the successful application of the synthetic hybrid-scale fiber matrix in various wound etiologies. The positive results observed suggest that the hybrid-scale fiber matrix can promote healing and may be a viable option for the treatment of difficult-to-heal wounds.
